# Oncogenic H-Ras Up-Regulates Acid β-Hexosaminidase by a Mechanism Dependent on the Autophagy Regulator TFEB

**DOI:** 10.1371/journal.pone.0089485

**Published:** 2014-02-24

**Authors:** Lorena Urbanelli, Alessandro Magini, Luisa Ercolani, Krizia Sagini, Alice Polchi, Brunella Tancini, Alessandro Brozzi, Tatiana Armeni, Giovanni Principato, Carla Emiliani

**Affiliations:** 1 Department of Experimental Medicine and Biochemical Sciences, University of Perugia, Perugia, Italy; 2 Department of Medical and Biological Sciences (DSMB), University of Udine, Udine, Italy; 3 Department of Clinical Sciences, Section of Biochemistry, Biology and Physics, Marche Polytechnic University, Ancona, Italy; 4 Centro di Eccellenza sui Materiali Innovativi Nanostrutturati (CEMIN), University of Perugia, Perugia, Italy; University of Saarland Medical School, Germany

## Abstract

The expression of constitutively active H-RasV12 oncogene has been described to induce proliferative arrest and premature senescence in many cell models. There are a number of studies indicating an association between senescence and lysosomal enzyme alterations, e.g. lysosomal β-galactosidase is the most widely used biomarker to detect senescence in cultured cells and we previously reported that H-RasV12 up-regulates lysosomal glycohydrolases enzymatic activity in human fibroblasts. Here we investigated the molecular mechanisms underlying lysosomal glycohydrolase β-hexosaminidase up-regulation in human fibroblasts expressing the constitutively active H-RasV12. We demonstrated that H-Ras activation increases β-hexosaminidase expression and secretion by a Raf/extracellular signal-regulated protein kinase dependent pathway, through a mechanism that relies on the activity of the transcription factor EB (TFEB). Because of the pivotal role of TFEB in the regulation of lysosomal system biogenesis and function, our results suggest that this could be a general mechanism to enhance lysosomal enzymes activity during oncogene-induced senescence.

## Introduction

The endosomal-lysosomal pathway consists of a dynamic system of organelles working to recycle cellular ingredients, thereby providing a constant supply of basic components necessary to maintain the health of the cell. Lysosomes contain over 80 hydrolytic enzymes including acidic glycohydrolases and proteases. Although they have been considered for a long time a terminal degradative compartment for turning over and recycling cellular constituents, it is now clear that they are also responsible for specific functions such as selective degradation of proteins, repair of the plasma membrane and release of cellular material [1], [2].

There is a link between H-Ras oncogene and the altered expression and subcellular distribution of lysosomal proteases such as cathepsins [3], [4], and even between H-Ras and lysosomal organelle density, distribution and ultrastructure [5]. H-Ras is a member of the small GTPase superfamily of proteins that function as molecular switches to transmit extracellular signals inside the cell and it is frequently mutated in different human cancers [6], [7]. H-Ras exerts its effect through the activation of a spectrum of downstream effectors mediating cytoplasmic signaling pathways [8]. The most studied Ras effector pathways are the Raf/extracellular signal-regulated protein kinase (ERK) cascade, the phosphatidylinositol 3-kinases (PI3Ks) [9] and the guanine nucleotide exchange factor (GEF) for the Ral small GTPase [10].

In primary fibroblasts the expression of the constitutively active H-RasV12 mutant is known to induce proliferative arrest and premature senescence, a state usually described as Oncogene Induced Senescence (OIS), which provides an intrinsic barrier to tumor development [11]. OIS requires activation of the p19Arf-p53 and p16Ink4a-Rb tumor suppressor pathways, and ablation of either pathways leads to cell immortalization [12]. Interestingly, one of the most frequently used biomarkers for cellular senescence is the so-called senescence associated β-galactosidase (SA-β-gal) [13], which is encoded by GLB1, the lysosomal β-galactosidase gene [14]. This evidence indicates an association between senescence and lysosomal enzymes alterations. Moreover, we previously observed that constitutively active H-RasV12 leads to an up-regulation of lysosomal glycohydrolases enzymatic activity in human fibroblasts [15].

Among lysosomal glycohydrolases, β-hexosaminidase (Hex, E.C.3.2.1.52) cleaves off terminal β-linked GlcNAc or GalNAc residues from oligosaccharides, glycolipids, glycoproteins and glycosaminoglycans. Two major lysosomal isoenzymes exist in human tissues which are the products of the assembly of two subunits, α and β, encoded by two closely related genes, HEXA and HEXB [16], [17]. The two isoenzymes Hex A (αβ) and Hex B (ββ) are both able to hydrolyze several natural and artificial substrates, but only Hex A can hydrolyze GM2 ganglioside, a glycosphingolipid which is an ubiquitous component of the external leaflet of the plasma membrane. Minor forms of β-hexosaminidase, such as the homodimer αα (Hex S) have been also characterized [18]. A fully processed Hex A has been found to be associated to the external leaflet of the plasma membrane as well as to the lysosomal membrane [19], specifically within lipid microdomains [20]. Recently, it was shown that the activation of TFEB, a transcription factor that controls lysosomal biogenesis and function, is accompanied by an increase of mature β-hexosaminidase on cell surface [21]. From a pathological point of view, mutations in the α- and β-subunit coding genes lead to the development of Tay-Sachs and Sandhoff diseases, respectively, which are severe lysosomal storage disorders associated with neurodegeneration [22]. In addition, β-hexosaminidase altered expression has been often associated with cancer [23], [24] and namely the presence of Hex S has been observed in leukaemic cells but not in their normal counterparts [25].

To gain insight into the association between OIS and lysosomal system alterations, we investigated the molecular mechanisms underlying lysosomal glycohydrolase β-hexosaminidase regulation in human fibroblasts expressing constitutively active H-Ras mutants and demonstrated that H-Ras activation increases β-hexosaminidase expression and secretion through a Raf/ERK dependent pathway involving TFEB, indicating a general mechanism to enhance lysosomal activity during OIS.

## Materials and Methods

### Cell Lines

HuDE (human dermal fibroblasts) were purchased from the Istituto Zooprofilattico Sperimentale, Brescia, Italy. Cells were cultured in Dulbecco’s modified Eagle’s medium (DMEM) containing 10% (v/v) heat-inactivated fetal bovine serum (FBS, Biokrom, Berlin, Germany), 2 mM glutamine, 100 U/ml penicillin, 100 µg/ml streptomycin and grown at 37°C in a 5% CO_2_. Cell viability was estimated by examining their ability to exclude trypan blue 0.1% (w/v) in 0.9% (w/v) NaCl. Cell morphology analysis by light microscopy was performed with a Nikon Eclipse TE2000U microscope.

### Ras Mutants, TFEB Expression and RNAi

Expression vectors encoding mutants of H-RasV12 have been previously described [26]. Vectors expressing H-RasV12, H-RasV12S35, H-RasV12G37 and H-RasV12C40 were obtained by subcloning H-Ras expressing pBABE-PURO retroviral vector [27] into the EcoRI site of pcDNA6 (Invitrogen, Carlsbad, USA) vector. Full-length human TFEB was cloned into KpnI/EcoRI sites of pcDNA6 as previously described [21]. shRNA expression constructs targeting TFEB were purchased from Origene (Rockville, USA). Scrambled shRNA (Origene) was used as control. Cells were transfected using Lipofectamine LTX (Invitrogen). Transiently transfected fibroblasts were selected using 4 µg/ml Blasticidin-S (Invitrogen) for 5 days or 0.3 µg/ml puromycin (Sigma) for 2–3 days.

### Preparation of Cell Lysates and Enzymatic Assays

Cell samples were washed twice with 0.9% (w/v) NaCl (500×g for 10 min) and suspended in 10 mM sodium phosphate pH 6.0 buffer and 0.5% (v/v) Nonidet P40 detergent. After 1 h incubation, they were vortexed and centrifuged at 16,000 g for 20 min. All procedures were carried out at 4°C. Supernatants were recovered and used for enzyme assays. Enzymatic activity of β-D-hexosaminidase was measured using 3 mM solutions of synthetic fluorogenic substrates 4-methylumbelliferyl-β-N-acetylglucosaminide (MUG) and 4-methylumbelliferyl-β-N-acetyl-glucosaminide-6-sulfate (MUGS) for total β-D-hexosaminidase and β-D-hexosaminidase A respectively. The substrates 4-methylumbelliferyl-α-D-mannoside (MU-α-mann) and 4-methylumbelliferyl-β-D-galactopyranoside (MU-β-gal) were used to deteminine β-D-galactosidase and α-D-mannosidase enzymatic activity, respectively. Assays were carried out in 96-well black multiplates (Greiner, Frickenhausen, Germany). At the end of the reaction period, 0.290 ml of 0.4 M glycine-NaOH buffer, pH 10.4 were added. Fluorescence of the liberated 4-methylumbelliferone was measured on a Infinite F200 fluorimeter (Tecan, Mannedorf, Switzerland) at 360 nm excitation, 450 nm emission. One unit is the amount of enzyme that hydrolyses 1 µmol of substrate/min at 37°C. Protein content was determined by the Bradford method, using bovine serum albumin as standard. Specific activity was expressed as enzyme units/mg of protein. To determine the enzymatic activity in cell culture medium, cells were washed and fresh medium added 16 hrs before the assay. Then medium was collected and, to eliminate cell debris, centrifuged at maximum speed in bench centrifuge. Supernatants were directly used for enzymatic activity determination as previously described. Values are given in mU/10^6^ cells.

### Preparation of Nuclear Extract

Cells (1×10^6^) were trypsinized and washed twice with cold PBS, lysed with 500 µl of lysis buffer (10 mM HEPES pH 7.9, 1.5 mM MgCl2, 10 mM KCl, 0.5 mM DTT, including as protease inhibitors 100 µg/ml aprotinin, 5 µg/ml leupeptin, 1 µg/ml pepstatin, 0.5 mM PMSF) and kept at 4°C for 15 min. Nuclei were pelleted at 6500 g for 1 min, and supernatants discarded. Pelleted nuclei were resuspended in 300 µl of extraction buffer (10 mM HEPES pH 7.9, 1.5 mM MgCl2, 420 mM NaCl, 0.2 mM EDTA, 25% v/v glycerol, including as protease inhibitors as above) and kept on ice for 30 min. Debris were pelleted at 12000 g for 10 min at 4°C, and supernatants recovered. Protein concentration was determined by Bradford assay, and samples were diluted to 1 µg/µl with extraction buffer. Nuclear proteins were stored at −70°C.

### Immunoblotting

Proteins (20–30 µg) were electrophoresed on acrylamide gel at 150 V for 1 h and transferred to PVDF membrane at 100 V for 1 h. Rabbit polyclonal anti-H-Ras antibody was purchased from Santa Cruz Biotechnology (Santa Cruz, USA), goat polyclonal anti-TFEB antibody was purchased from Abcam (Cambridge, UK). As internal control, membrane was probed with mouse monoclonal anti β-actin (Sigma-Aldrich, St Louis, USA) and nuclear extracts were normalized with rabbit polyclonal anti-H3 antibody (Millipore, Billerica, USA). Sheep anti-goat (Sigma), donkey anti-rabbit and sheep anti-mouse HRP-linked secondary antibodies (GE Biosciences, Piscataway, USA) were used according to manufacturer’s instructions. Immunoblots were detected by chemiluminescence using ECL (GE Biosciences).

### Quantitative PCR

RNA was extracted using Trizol reagent (Sigma) according to the manufacturer’s instructions. One microgram of RNA was reverse-transcribed into cDNA using random hexamers and SuperScript II Reverse Transcriptase (Invitrogen, Carlsbad, CA, USA). cDNA was used to determine HEXA, HEXB and TFEB transcripts by quantitative RT-PCR (qRT-PCR) in a Stratagene Mx3000P Q-PCR machine (Agilent Technologies, La Jolla, USA). Reactions were performed in triplicate using Brilliant II SYBR Green Q-PCR Master Mix (Agilent Technologies). Sequences used for the amplification were 5′-GCA TTT GAA GGT ACC CCT GA (forward) and 5′-TCA ACT TGT TGC TCC ACA GC (reverse) for HEXA, 5′-TTG GGA GGA GAT GAA GTG G (forward) and 5′-AAA CCT CCT GCC AGA CAA TG (reverse) for HEXB, 5′-TGA TCC CCA AGG CCA ATG AC (forward) and 5′-TCC CTG GAC TTT TGC AGG TC (reverse) for TFEB. β-Actin (ACTB) or GADPH genes were amplified as endogenous control using primers 5′-AGA AAA TCT GGC ACC ACA CC (forward) and 5′-GGG GTG TTG AAG GTC TCA AA (reverse) or 5′-GAG AAG GCT GGG GCT CAT TT (forward) and 5′-AGT GAT GGC ATG GAC TGT GG (reverse) respectively. Data were analyzed using the ΔΔCt method. ΔCt was calculated subtracting the average Ct value of ACTB or GADPH to the average Ct value of HEXA or HEXB gene for each sample. ΔΔCt is the difference between the ΔCt for each samples and the ΔCt of mock transfected fibroblasts as control. The reported fold expression, expressed as RQ (relative quantity), was calculated by 2^−ΔΔCt^.

### Construction of Luciferase Reporter Vectors and Reporter Gene Assay

The promoter region of human HEXA and HEXB genes were selected by searching the Human Genome Resources available at NCBI. The nucleotide upstream of the translation start site was numbered −1. Genomic DNA was prepared from human primary fibroblasts using QIAmp method (Qiagen, Hilden, Germany). Reporter gene constructs containing the 5′-flanking region of HEXA and HEXB were obtained by PCR amplification of the human genomic DNA using high fidelity DNA polymerase Expand Long (Roche, Penzberg, Germany). For HEXA gene promoter, PCR was performed with primers HEXA.rev, mapping at +9 bp with respect to the first ATG (5′-CCC AAG CTT GGG CTT GTC ATG GCC CGC TGG TC) and HEXA.for (5′-CCC AAG CTT GGG CGG GTG AGC TGT CTA GTT CC), mapping at −1594 bp, both containing the HindIII restriction site. For HEXB gene promoter, PCR was performed with primers HEXB.rev, mapping +15 bp downstream the first ATG (5′- GGG GTA CCC CGC ACA GCT CCA TGG CC) and HEXB.for (5′- GGG GTA CCC CGA AAG GCA GGA GAA GGT CAG), mapping at −1570 bp, both containing the KpnI restriction site. HEXA and HEXB PCR amplicons were subcloned into the firefly luciferase reporter vector pGL3 Basic (Promega, Madison, USA), obtaining the pGL3-HEXA(−1594/+9 bp) and the pGL3-HEXB (−1570/+15 bp) constructs.

HEXA 5′ deletions were generated by BglII/HindIII digestion (−891/+9 bp) and XhoI/HindIII digestion (−312/+9 bp) of the HEXA(−1594/+9 bp) construct. Further 5′ deletions were generated from pGL3-HEXA(−1594/+9 bp) construct by PCR with the following forward primers: 5′- CCC AAG CTT GGG TGT GGG TCC TCC TGG GG (−136/+9 bp), 5′-CCC AAG CTT GGG CCT CTG GTC ACG TGA TTC G (−100/+9 bp) and 5′-CCC AAG CTT GGG ATA AGT CAC GGG GGC GCC G (−78/+9 bp). HEXB 3′ deletions were generated by HindIII/KpnI digestion (−1570/−717 bp) and XhoI/KpnI digestion (−1570/−193 bp) of the pGL3-HEXB(−1570/+15 bp) construct. A series of 5′ deletions were generated by PCR from the pGL3-HEXB(−1570/+15 bp) construct with the following forward primers: 5′-GGG GTA CCC CTG GAC AGG GCG GGC TG (−183/+15 bp), 5′-GGG GTA CCC CGA GGA CGC TCC CGG GGC (−163/+15 bp), 5′-GGG GTA CCC CGT CGG GGG CGG GCG CG (−132/+15 bp) and 5′- GGG GTA CCC CTC GGT GAC TCA CCC GCG G (−105/+15 bp). Mutation of HEXA promoter sequences was obtained with modified oligonucleotide 5′- CCC AAG CTT GGG CCT CTG GTC GTT TGA TTC GCC GAT AAG HEXA.mut (−100/+9). Mutagenic exchange was confirmed by sequencing.

HuDe cells (1.7×10^5^) were seeded in 6-well plates, cultured for 24 hrs and transfected using 5 µl of Lipofectamine (Invitrogen). Transfections were performed with 900 ng of pGL3 vector constructs and 100 ng of pRL-SV40 (Promega, Fitchburg, USA) for transfection efficiency control. After 48 hrs, cells were washed with PBS and harvested in Reporter Lysis Buffer (Promega). Cell extracts were centrifuged at 12000 g for 10 min to pellet cell debris and supernatant recovered for assays. Quantification of firefly and Renilla luciferase activities was performed with the Dual Luciferase Reporter Assay System (Promega). The relative firefly luciferase activity was calculated by normalizing transfection efficiency.

For co-transfections with H-Ras, TFEB or shRNA 880 ng of vectors encoding for H-Ras mutants, TFEB or shRNA were mixed with 100 ng of pGL3 vector constructs and 20 ng of pRL-SV40 vector (Promega) as transfection efficiency control.

### Bioinformatic Analysis

HEXA and HEXB gene sequences were obtained from National Center for Biotechnology Information under the Accession Number NG_009017.1 and 1.NG_009770.1, respectively. Putative transcription factor binding sites within the human regulatory region were analyzed by MatInspector software algorithms (Genomatix, Munich, Germany).

### EMSA

HEXA and HEXB promoter segments were synthesized by PCR using 5′ biotinylated oligonucleotides or alternatively annealed as complementary 5′ biotinylated oligonucleotides. PCR amplicons were purified with QIAquick PCR purification kit and quantified on 2% agarose gel. Gel-shift assays were performed using 5–10 fmol of biotinylated segment and 1–2 µg of nuclear extract per assay in a final volume of 20 µl. The presence of poly(dI/dC) prevented non specific protein-DNA binding. Incubation for 20 min at RT preceded electrophoretic separation on a native 4–6% polyacrylammide gel in 0.5X TBE. Probes were transferred on nylon membrane (Hybond, Escondido, USA) and incubated with streptavidin conjugated with horseradish peroxidase and developed with luminol. All reagents were purchased from LightShift Chemiluminescent EMSA Kit (Pierce, Rockford, USA). For supershift analysis, appropriate amounts of anti-TFEB or anti-USF antibodies (SantaCruz) were added to samples and pre-incubated on ice for 20–30 min before the addition of biotinylated segments, then incubated for 20 min at RT. The promoter fragment annealed and used for the characterization of protein binding was HEXA (−100/−70 bp).for 5′-GAG CCG CCT CTG GTC ACG TGA TTC GCC GAT AAG TC and HEXA (−100/−70 bp).rev 5′-GAC TTA TCG GCG AAT CAC GTG ACC AGA GGC GGC TC.

### Chromatin Immunoprecipitation (ChIP) Assay

ChIP assays were performed using the Thermo Scientific Pierce Agarose ChIP Kit (Thermo Scientific Pierce, Rockford, IL, USA). Cells were fixed with 1% formaldehyde in PBS for 10 min, washed twice with ice-cold PBS and resuspended in lysis buffer. Nuclei were recovered by centrifugation, then MNase digestion was carried out at 37°C for 15 min. Nuclei were lysed and extracts were immunoprecipitated overnight at 4°C using 4 µg antibody against TFEB. Normal rabbit IgG was used as a negative control. Immune complexes were collected with protein A agarose and washed, then cross linking between proteins and DNA was reversed by incubation at 65°C for 40 min. Protein-bound DNA was recovered using affinity chromatography purification columns according to the manufacturer’s protocol, and 5 µl of lysed nuclei were also purified with the same procedure and used as input. DNA amplification was performed by PCR using the as primers for HEXA promoter 5′-CAA TCC GCT GCA CGT AGC AGG -3′ (forward) and 5′-TGG CCA CGT GAG ACC CTG GTC -3′ (reverse) (−189/−30 bp) and for HEXA exon 11 as negative control 5′-ATT CAG CCA GAC ACA ATC ATA CAG -3′ (forward) and 5′-GCC AGG GGT TCC ACT ATG TAG -3′ (reverse). Amplification products were separated on a 2% agarose gel.

### Statistical Analysis

All values are reported as mean ± s.d. Statistical comparison of differences among groups of data was carried out using Student’s t-test. P-values ≤0.05 were considered significant (*), P-values ≤0.01 were considered highly significant (**).

## Results

### Lysosomal Glycohydrolases Level in HuDe Fibroblasts Expressing H-Ras Mutants

**Figure 1:**
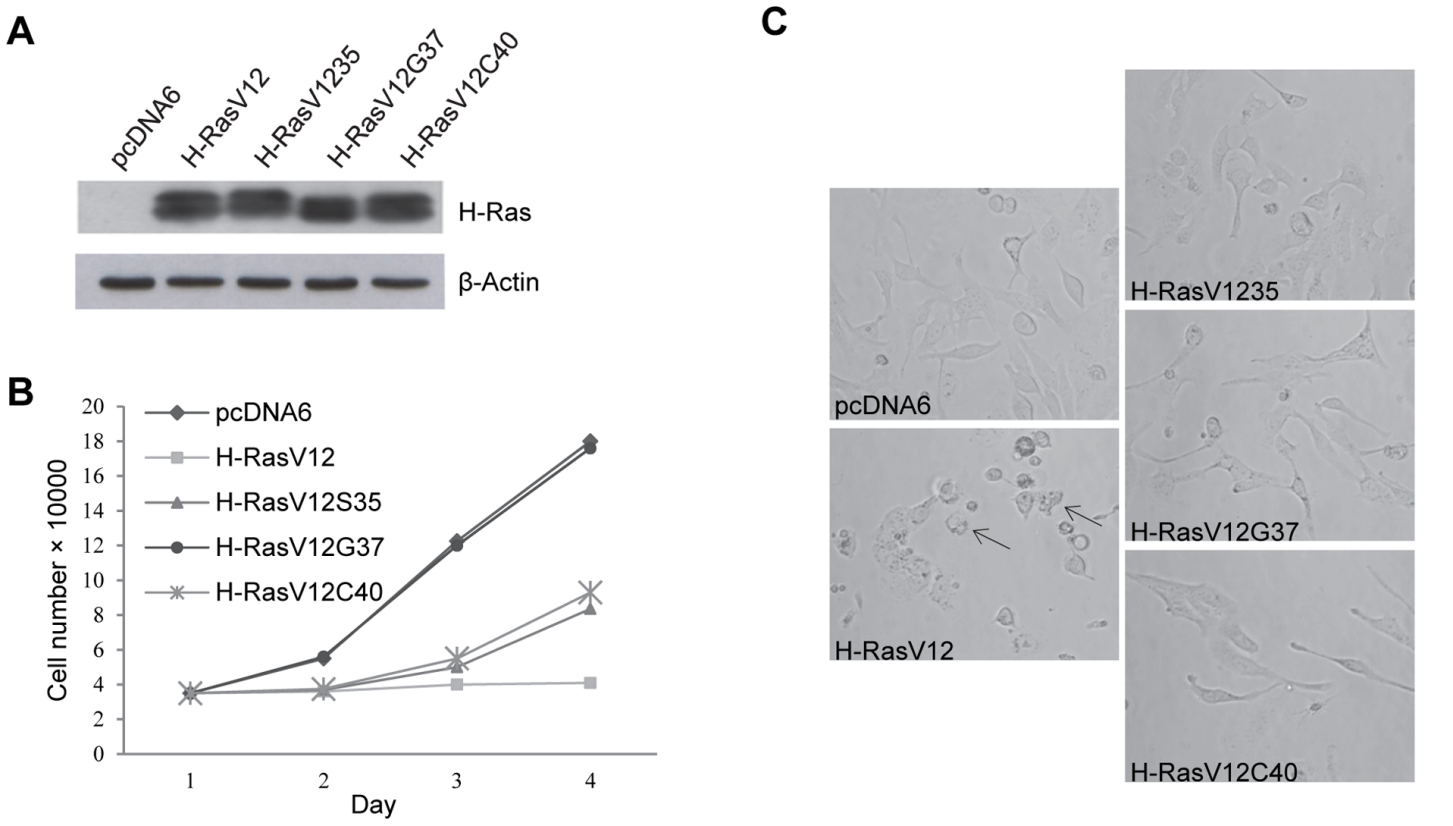
Analysis of HuDe fibroblasts transfected with H-RasV12, H-RasV12S35, H-RasV12G37, H-RasV12C40 mutants. A, Immunoblotting analysis. Cell extracts were incubated with an anti-H-Ras antibody. As internal control an anti-β actin antibody was used. The increased expression of H-Ras with respect to empty vector transfected cells is shown. B, Growth curve. Cells were counted after the end of the selection with blasticidin-S (4 µg/ml) and cell number is reported. C, Cells morphology was examined by light microscopy, 200× total magnification. Arrows indicate flattened cells and cytosolic vacuolization associated with senescence.

To investigate the role of H-Ras as an upstream modulator of lysosomal glycohydrolases in human dermal fibroblasts (HuDe), we transfected cells with the constitutively active H-RasV12 oncogene, using fibroblasts transfected with the empty vector as control. High levels of H-Ras were detected by immunoblotting (Fig. 1A). Expression of constitutively active H-RasV12 has been previously reported to lead to an arrest of cell proliferation [28] and in our cell model H-RasV12 actually induced an arrest of cell proliferation (Fig. 1B) and cell morphological alterations (Fig. 1C) indistinguishable from cellular senescence, as we have also previously reported [29]. This result clearly indicated that tumor suppressor pathways were not ablated and cells could not escape from H-Ras induced senescence, an intrinsic barrier to tumor development [30]. As H-Ras regulates many downstream effector pathways, to gain insight which of them could be responsible for modulating lysosomal glycohydrolases, we also expressed three Ras double mutants (H-RasV12S35, H-RasV12G37, H-RasV12C40). These mutants are all constitutively active, but possess an additional mutation impairing their interaction with downstream H-Ras effectors. Nevertheless, they retain specific activating properties: the H-RasV12S35 mutant binds and activates Raf but is reduced in PI3K and Ral-GEF binding, the RasV12G37 mutant binds and activates Ral-GEF but is reduced in its ability to bind to Raf or PI3K and the RasV12C40 mutant binds and activates PI3K but is reduced in Raf and Ral-GEF binding [26], [31]. High levels of H-Ras double mutants expression were detected by immunoblotting (Fig. 1A). The expression of H-RasV12S35 and H-RasV12C40 double mutants impaired cell growth (about 50% with respect to control cells after 72 hrs), thus indicating that the activation of Raf/ERK and PI3K signaling pathways is relevant for cell-cycle arrest during OIS (Fig. 1B) [32]. The expression of H-RasV12G37 double mutant did not alter cell growth, which was indistinguishable from that of control cells (Fig. 1B), thus indicating that in our cell model the activation of Ral-GEF signaling pathway is less relevant for cell cycle arrest. Although cell growth was affected by H-RasV12S35 and H-RasV12C40 mutants expression, their morphology remained similar to that of cells transfected with either pcDNA6 as control or H-RasV12G37 mutant (Fig. 1C), even if subtle morphological changes could not be excluded.

The enzymatic activity of lysosomal glycohydrolases was determined in H-Ras expressing fibroblasts by measuring their ability to hydrolyze synthetic substrates: β-hexosaminidase (Hex) activity was measured using the two substrates MUG, which is hydrolyzed by both α- and β-subunit forming Hex isoenzymes (Total Hex), and MUGS, which is hydrolyzed only by the α-subunit-containing isoenzyme (Hex A). β-galactosidase (β-gal) and α-D-mannosidase (α-man) activities were assayed using MU-β-gal and MU-α-mann as substrates, respectively. Results reported in Table 1 showed that in the presence of H-RasV12, Hex A and Total Hex enzyme activities towards both MUGS and MUG were not significantly affected, whereas α-man and β-gal enzymatic activity were increased.

**Table 1:** Glycohydrolases specific activity in cell extracts of HuDe fibroblasts transfected with H-Ras mutants.

	pcDNA6	H-RasV12	H-RasV12S35	H-RasV12G37	H-RasV12C40
**HexA**	4.90±0.10	4.45±0.13	5.27±0.09	4.90±0.03	4.70±0.21
**Total Hex**	45.9±1.6	40.2±1.2	49.2±1.5	44.7±0.5	41.3±0.9
**β-gal**	1.27±0.05	2.16±0.05**	1.66±0.04*	1.34±0.01	1.27±0.01
**α-man±**	0.263±0.006	0.366±0.002**	0.256±0.001	0.267±0.010	0.229±0.011

Enzyme activity is expressed as mU/mg of protein (specific activity). Each value is the mean±s.d of at least three experiments, each one in duplicate.

*P<0.05;

**P<0.01 vs empty vector.

In normal cells, about 5–20% of newly synthesized lysosomal proteins escape binding to mannose phosphate receptors and become secreted [33]. To assess the level of secreted lysosomal glycohydrolases, we measured their enzymatic activity in the culture medium of fibroblasts expressing H-Ras mutants. β-gal and α-man enzymatic activity could not be determined (data not shown) but Hex A enzymatic activity was increased (about 3-fold with respect to empty vector transfected cells), and Total Hex enzymatic activity was also significantly increased (Table 2). We concluded that H-RasV12 expression leads to higher levels of Hex secreted isoenzymes. In addition, the secretion was mainly dependent on the activation of the Raf/ERK pathway, as shown by H-RasV12S35 mutant activity.

**Table 2:** Glycohydrolases specific activity in the cell culture medium of HuDe fibroblasts transfected with H-Ras mutants.

	pcDNA6	H-RasV12	H-RasV12S35	H-RasV12G37	H-RasV12C40
**HexA**	0.569±0.019	1.444±0.023**	1.100±0.026**	0.671±0.018*	0.591±0.022
**Total Hex**	4.15±0.16	7.27±0.22**	7.84±0.29**	4.98±0.12*	3.89±0.05

Enzyme activity is expressed as mU/10^6^ cells. Each value is the mean±s.d. of at least three experiments, each one in duplicate.

*P<0.05;

**P<0.01 vs empty vector.

**Figure 2:**
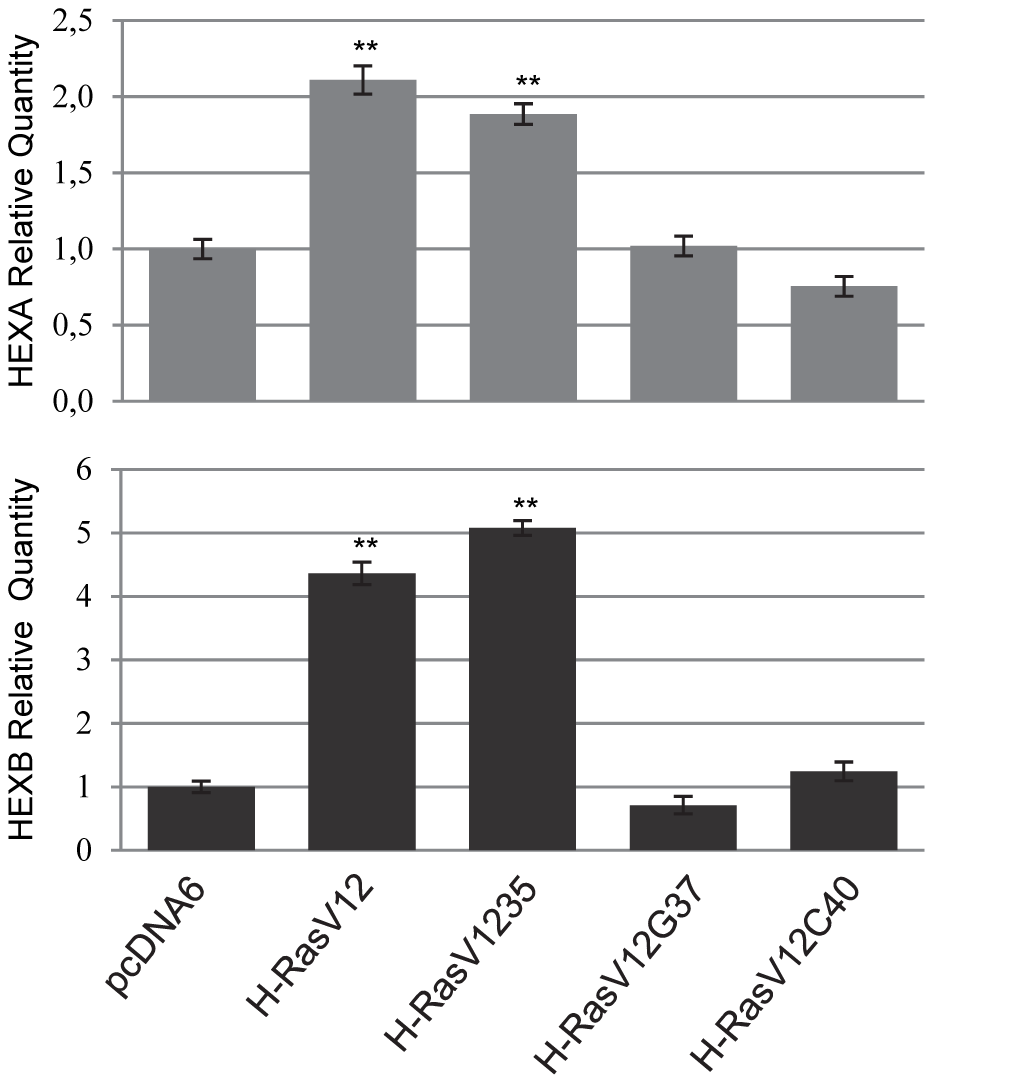
HEXA and HEXB gene expression analysis by qRT-PCR. About 10-Ras mutants, with respect to those infected with the vector alone as control, is represented. The value is expressed as Relative Quantity (RQ). Analysis was repeated three times. The mean±s.d. of a representative experiment is reported. ** P<0.01 vs empty vector.

To investigate the molecular mechanism underlying the increased level of Hex isoenzymes upon H-Ras mutants expression, we determined the level of HEXA and HEXB gene transcripts by qRT-PCR. We detected an increase of both transcripts, that was about 2-fold for HEXA and about 4-fold for HEXB (Fig. 2). Besides, the pathway responsible for the increased expression was that dependent on the Raf/ERK pathway, as shown by H-RasV12S35 mutant results. These findings clearly indicated that the increase of Hex isoenzymes activity in cell culture medium was due to a transcriptional up-regulation.

### Regulatory Sequences within the Human HEXA and HEXB Promoters

To shed light on the influence of constitutively active H-Ras mutants on Hex isoenzymes expression, we investigated the regulation of HEXA and HEXB gene promoters. First, we determined the minimal promoters of both genes. According to Norflus et al. [34], preliminary results in mouse cells located HEXA promoter within 100 bp from the first ATG and HEXB promoter within 150 bp from the first ATG. We constructed deletion mutants of the human HEXA and HEXB gene promoters in the reporter vector pGL3 Basic and transfected these constructs in HuDe fibroblasts to quantify luciferase activity.

**Figure 3:**
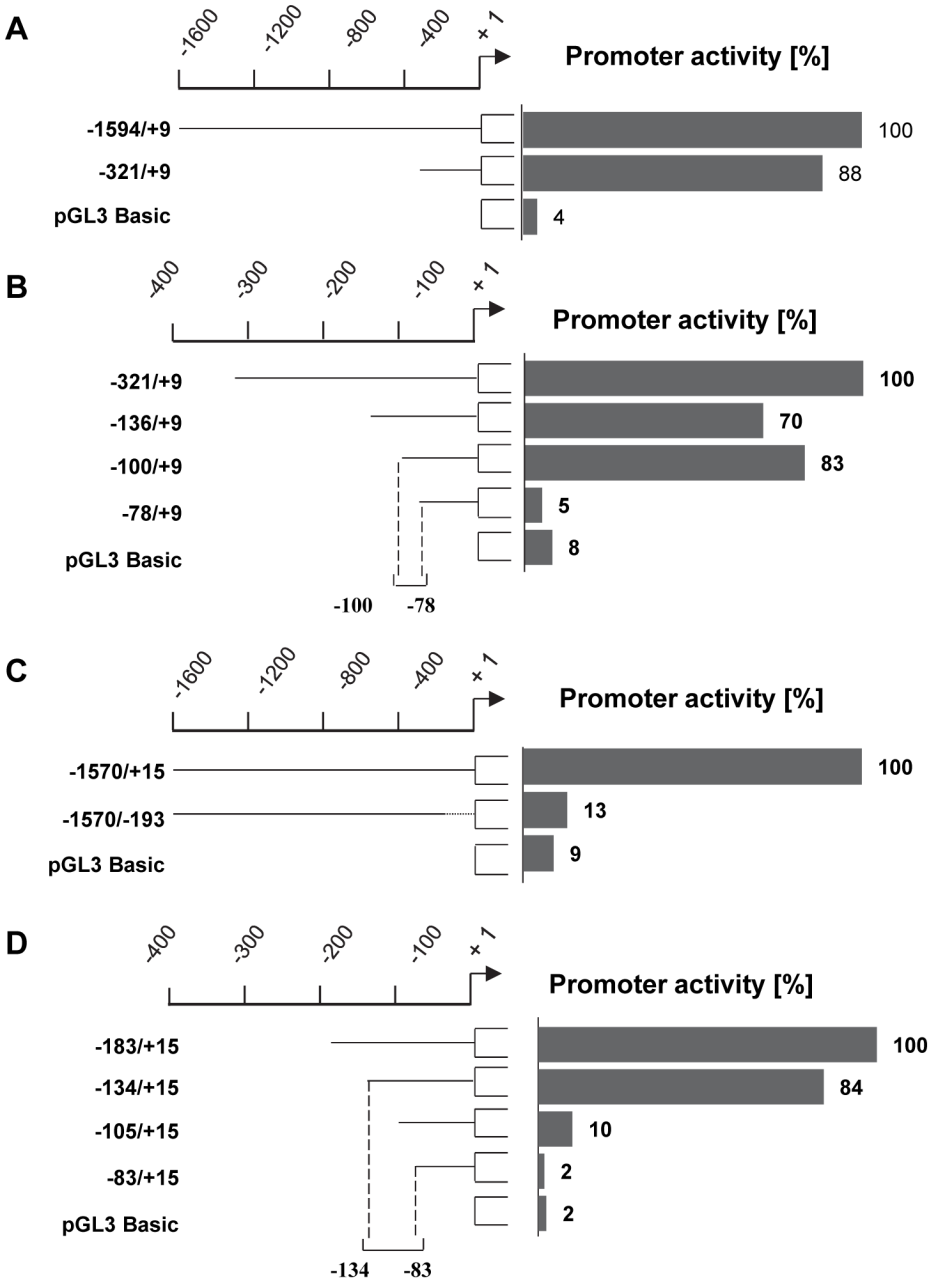
HEXA and HEXB gene promoter active segments. Deletions of lysosomal HEXA and HEXB promoters were tested in luciferase reporter assay. A and B, Promoter active segments of the (−1594/+9 bp) and (−321/+9 bp) deletion constructs of HEXA promoter. A, Promoter activity is given in percent of the segment −1594/+9 (set 100). B, Promoter activity is given in percent of HEXA −321/+9 segment (set 100). C and D. Promoter active segments of the (−1570/+15 bp) and (−183/+15 bp) deletion constructs of HEXB promoter. C, Promoter activity is given in percent of the segment −1570/+15 (set 100). D, Promoter activity is given in percent of the segment −183/+15 (set 100). Given are mean values of at least three separate assays, each conducted in duplicates. S.d. of duplicates and separate assays was below 15%.

In the case of HEXA promoter (Fig. 3 A and B), no significant reporter activity was detected with the construct containing the sequence −78/+9 with respect to the first ATG, in comparison with luciferase activity obtained with the pGL3 Basic vector alone as control. However, a strong reporter activity (about 10-fold) was detected when cells were transfected with the plasmid pGL3-HEXA(−100/+9 bp), and no additional increase of luciferase activity was observed when longer constructs were transfected. Of consequence, according to 5′ deletion results the promoter activity was located in a very short sequence between −78 and −100 with respect to the first ATG. In the case of HEXB promoter (Fig. 3 C and D), no significant reporter activity was detected with the construct containing the sequence −83/+15 with respect to the first ATG, while a strong reporter activity (about 5-fold) was detected with the plasmid pGL3-HEXB(−105/+15 bp) with respect to pGL3 Basic vector alone as control. Besides, a stronger luciferase activity was detected when cells were transfected with the plasmid pGL3-HEXB(−134/+15 bp), whereas no increase of luciferase activity was observed when longer constructs were transfected. Thus the 5′ deletion data located HEXB promoter activity to a 50 bp sequence between −134 and −83 with respect to the first ATG, indicating a more complex regulation of HEXB gene with respect to HEXA.

### Effect of Mutations on Human HEXA Promoter Activity

**Figure 4:**
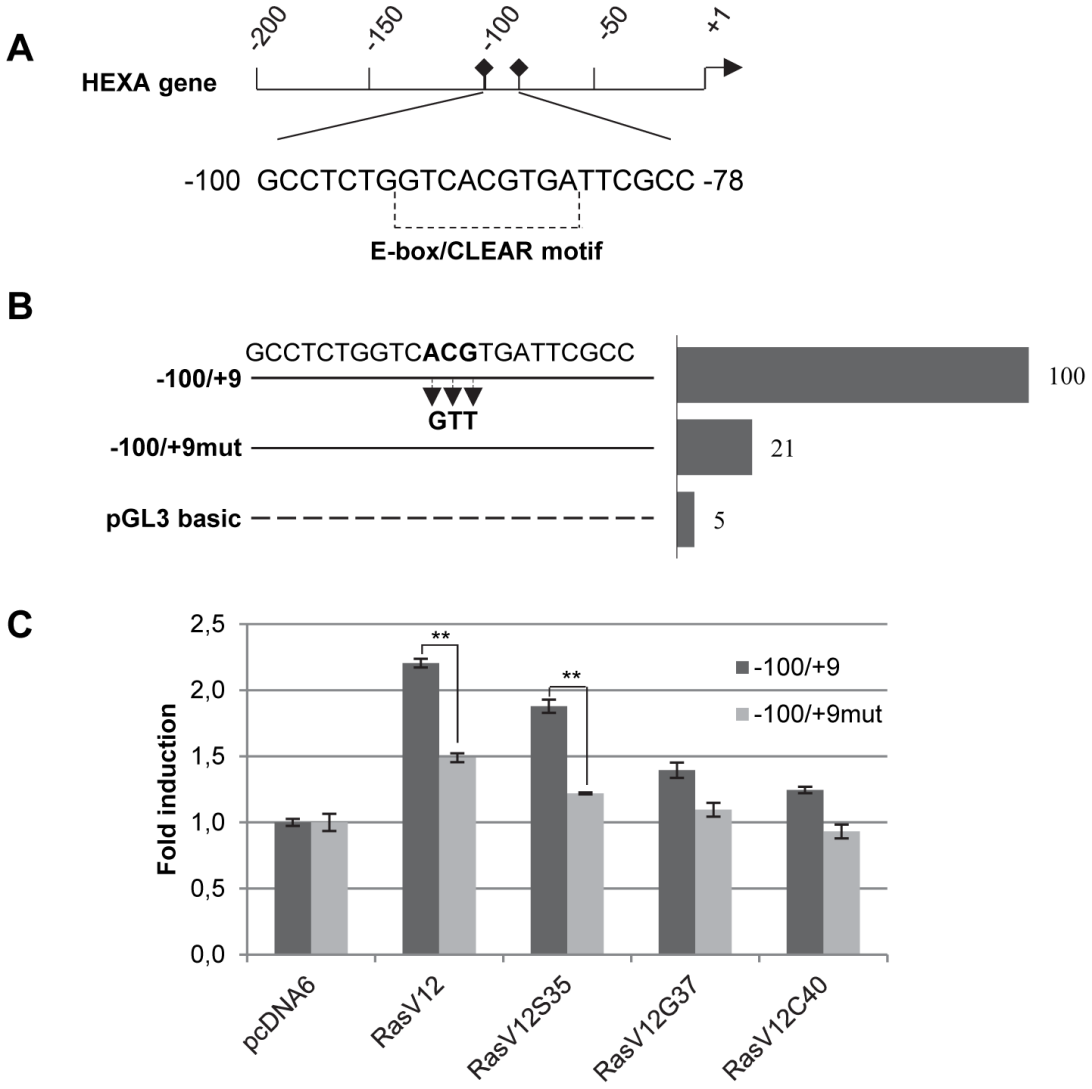
Identification of factors relevant for HEXA gene promoter activity. A, Sequence of the segment with the highest promoter activity −100/−78. The E-box/CLEAR motif is indicated. B, Reporter activity of the wild type sequence (set 100) compared with the sequence mutated in the E-box. Measures are mean of three separate assays, each performed in duplicate. S.d. of duplicates and separate assays was below 15%. C, Wild type and mutated segment −100/−78 co-transfected in HuDe fibroblasts with an excess of H-Ras mutants and the empty vector as control. Vertical bars indicate reporter activity fold induction of wild type and mutated constructs versus empty vector (set 1). Measures are mean values±s.d. of three separate assays, each one in duplicate. ** P<0.01 vs wild type sequence.

Sequence analysis of the human HEXA promoter using MatInspector revealed that in the segment −100/−78 is present an E-box (CANNTG), which is a well known target site for basic helix-loop-helix (bHLH) transcription factors. The E-box overlapped a CLEAR (Coordinated Lysosomal Expression and Regulation) motif (Fig. 4A), a palindromic 10 bp motif highly enriched in lysosomal gene promoters, which regulates the transcription of lysosomal genes [35]. Sequence analysis of HEXB gene also revealed a CLEAR motif located in the segment −134/−105, which showed the strongest promoter activity, but additional consensus elements were also present in the −105/−83 segment, which was also significantly active (data not shown).

HEXA promoter active sequence was therefore examined by mutational analysis (Fig. 4B). Three bases were exchanged within CLEAR motif by oligonucleotide-based mutagenesis to provide (−100/+9)mut segment. The reporter activity of this mutant was strongly affected, as it was decreased to 21% with respect to the wild-type, thus confirming that the CLEAR motif is of fundamental importance to drive the activity of HEXA promoter in HuDe cells.

To gain further evidence that HEXA gene transcript level is up-regulated by active H-Ras through a transcription factor binding to the CLEAR motif present in the −100/+9 promoter segment, we co-transfected the pGL3-HEXA(−100/+9 bp) construct with an excess of plasmid encoding H-Ras mutants, and pcDNA6 as control. Results showed (Fig. 4C) that the expression of H-RasV12 induced an increase of luciferase activity driven by the pGL3-HEXA(−100/+9 bp) construct, that was impaired when the pGL3-HEXA(−100/+9 bp)mut construct was used. Moreover, the increase of reporter activity was mediated by the Raf/ERK pathway, in correlation with enzymatic activity and transcript analysis results.

### Proteins Binding to Promoter Active Segment

**Figure 5:**
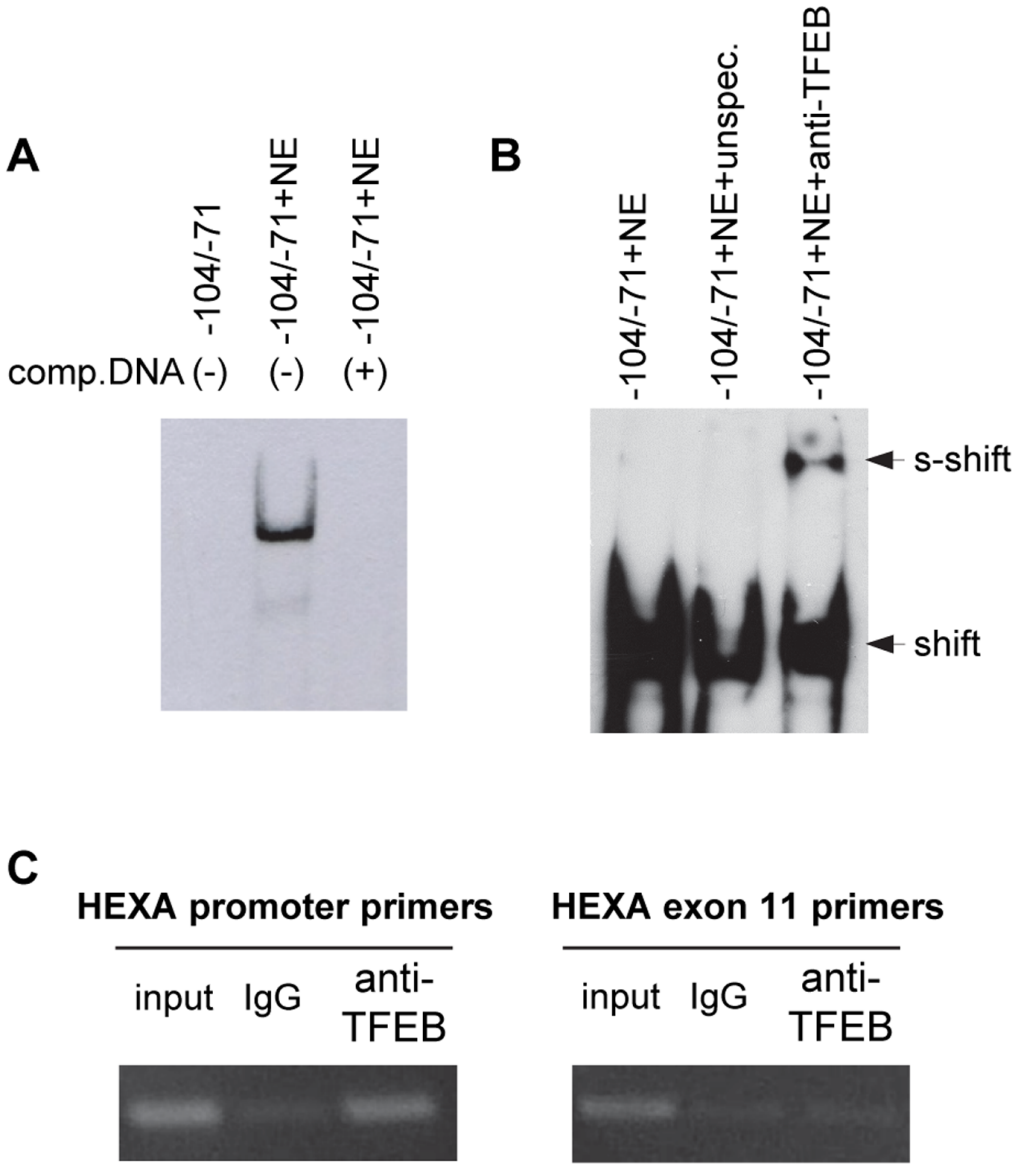
Binding of TFEB to the HEXA gene promoter *in vitro* and *in vivo*. A, Protein binding analysis by EMSA. Controls were run either without NE or with an additional 100-fold molar excess of unbiotinylated promoter segment as competitor DNA. B, Characterization of protein binding by super-shift analysis. HuDe NE was incubated with the segment −71/−104. The addition of NE, unspecific (anti-USF; 1 µg/assay) or specific antibody (anti-TFEB; 1 µg/assay) is indicated. C, ChIP assay using anti-TFEB or IgG control antibodies was performed on chromatin isolated from HuDe starved cells. An equivalent amount of chromatin was used as ‘input’ DNA. PCR products of the HEXA promoter region (left panel) and HEXA exon 11 control region (right panel) run on a 2% agarose gel are shown.

To assess the binding capability of proteins present in HuDe nuclear extract (NE), the region −104/−71 of the HEXA promoter was employed in EMSA. When incubated with NE, protein binding occurred within this segment (Fig. 5A). As the sequence analysis suggested that the CLEAR motif was a potential binding sites for TFEB, we carried out super-shift analysis in the presence of an anti-TFEB antibody and complexes binding to the segment containing the TFEB consensus sequence showed up-shifts in the presence of the anti-TFEB antibody (Fig. 5B). To test whether the TFEB binds to HEXA promoter *in vivo*, we performed chromatin immunoprecipitation (ChIP) using the anti-TFEB antibody and specific primers for the HEXA promoter region. The binding of transcription factor was specifically assessed, as only very low levels of PCR product were present in chromatin samples immuniprecipitated with non immune IgG (Fig. 5C, left panel). Besides, the specificity of ChIP was further demonstrated by the absence of binding to the HEXA exon 11 region (Fig. 5C, right panel).

### TFEB Drives HEXA Expression in vivo and its Nuclear Localization is Increased upon H-RasV12 Expression

**Figure 6:**
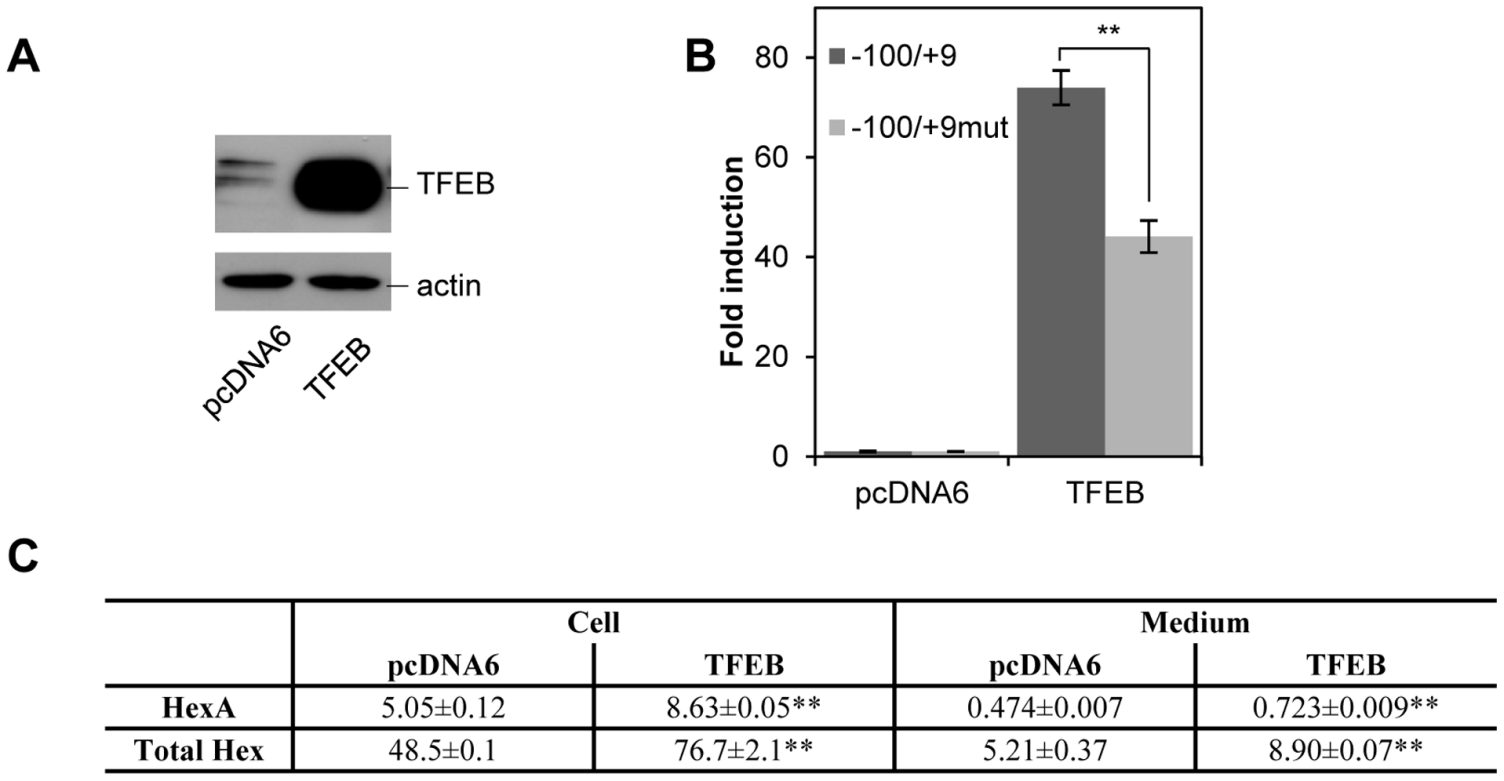
Analysis of HuDe fibroblasts over-expressing TFEB. A**,** Immunoblotting of cells transfected with TFEB. Extracts from cells transfected with TFEB or empty vector as control were incubated with an anti-TFEB antibody. As internal control, an anti-βactin antibody was used. B, Reporter activity of HEXA promoter in the presence of TFEB. The wild type and E-box mutated segments −78/−100 were co-transfected with an excess of TFEB expressing plasmid. Vertical bars indicate reporter activity fold induction in the presence of TFEB, with respect to empty vector (set 1). Measures are the mean ± s.d. of three separate experiments, each one in duplicate. C, Hex A and Total Hex enzymatic activity in cell extracts and culture medium of HuDe fibroblasts expressing TFEB. Results are indicated as mU/mg of proteins (specific activity) for cell extracts and in mU/10^6^ cells for cell culture medium. ** P<0.01.

**Figure 7:**
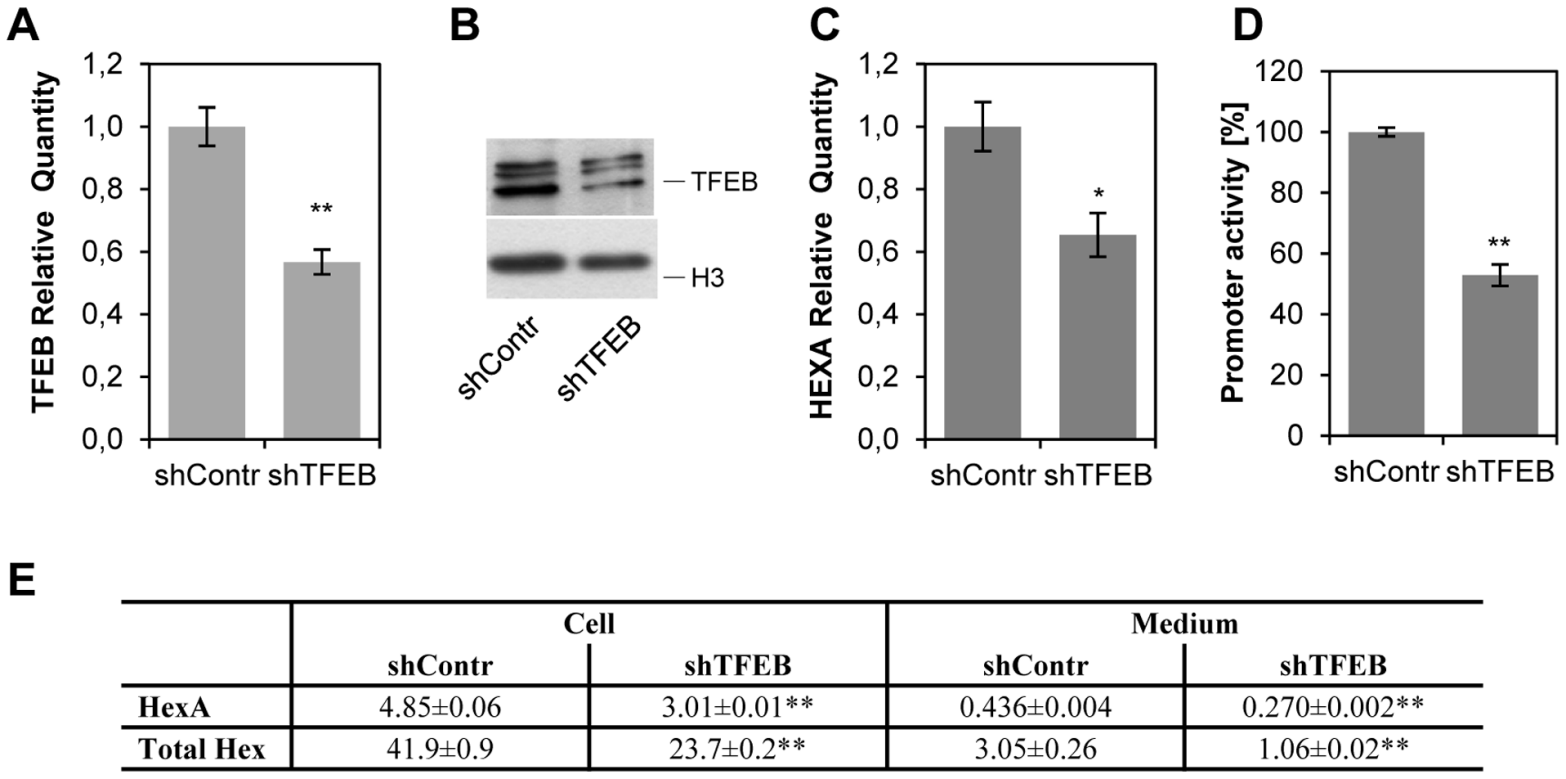
Down-regulation of HEXA gene expression by TFEB knock down. HuDe fibroblasts were transfected with shRNA for TFEB (shTFEB) or scrambled shRNA (shContr) as control. A, Analysis of TFEB transcript level by qRT-PCR. Reactions were performed using SYBR green, GADPH gene was used as endogenous control. The value is expressed as Relative Quantity (RQ). Each measure was repeated at least three times, each one in triplicate. The mean±s.d.of a representative experiment is reported. B, Analysis of TFEB expression by immunoblotting. Nuclear extracts were tested with an anti-TFEB antibody. As internal control, an anti-H3 histone antibody was used. C, Analysis of HEXA transcript level by qRT-PCR. Reactions were performed and elaborated as described in panel A. D, Reporter activity of HEXA promoter in TFEB knocked down cells. The segment −78/−100 (set 100) was co-transfected with an excess of shTFEB or shContr vector. Measures are the mean ± s.d. of three separate experiments, each one in duplicate. E, Hex A and Total Hex enzymatic activity in cell extracts and culture medium of HuDe transfected with shTFEB or shContr. Results are indicated as mU/mg of proteins (specific activity) for cell extracts and in mU/10^6^ cells for cell culture medium. * P<0.05; ** P<0.01.

To directly link Hex A and Total Hex level to TFEB, we over-expressed TFEB in HuDe fibroblasts. As shown in Fig. 6B, co-transfection of TFEB actually induced an increased of the luciferase activity driven by the pGL3-HEXA(−100/+9 bp) construct (about 70-fold with respect to the pGL3Basic alone), which was significantly reduced when TFEB was co-transfected with the pGL3-HEXA(−100/+9 bp)mut construct (about 40-fold), thus demonstrating that TFEB can modulate reporter activity by binding to the HEXA promoter active segment. In addition, Hex A and Total Hex enzymatic activity was measured in cell extracts and culture medium of TFEB expressing fibroblasts (Fig. 6C): an increase of Hex A and Total Hex activities was observed in both cases. Conversely, to test whether TFEB suppression led to β-hexosaminidase down regulation, HuDe cells were transfected with shRNA for TFEB. Down-modulation of the gene had effects on HEXA gene transcription (Fig. 7C) and HEXA promoter driven luciferase activity (Fig. 7D). Moreover, Hex A and Total Hex enzymatic activity was assayed both in cell extracts and culture medium (Fig. 7E), confirming a decrease in both cases.

**Figure 8:**
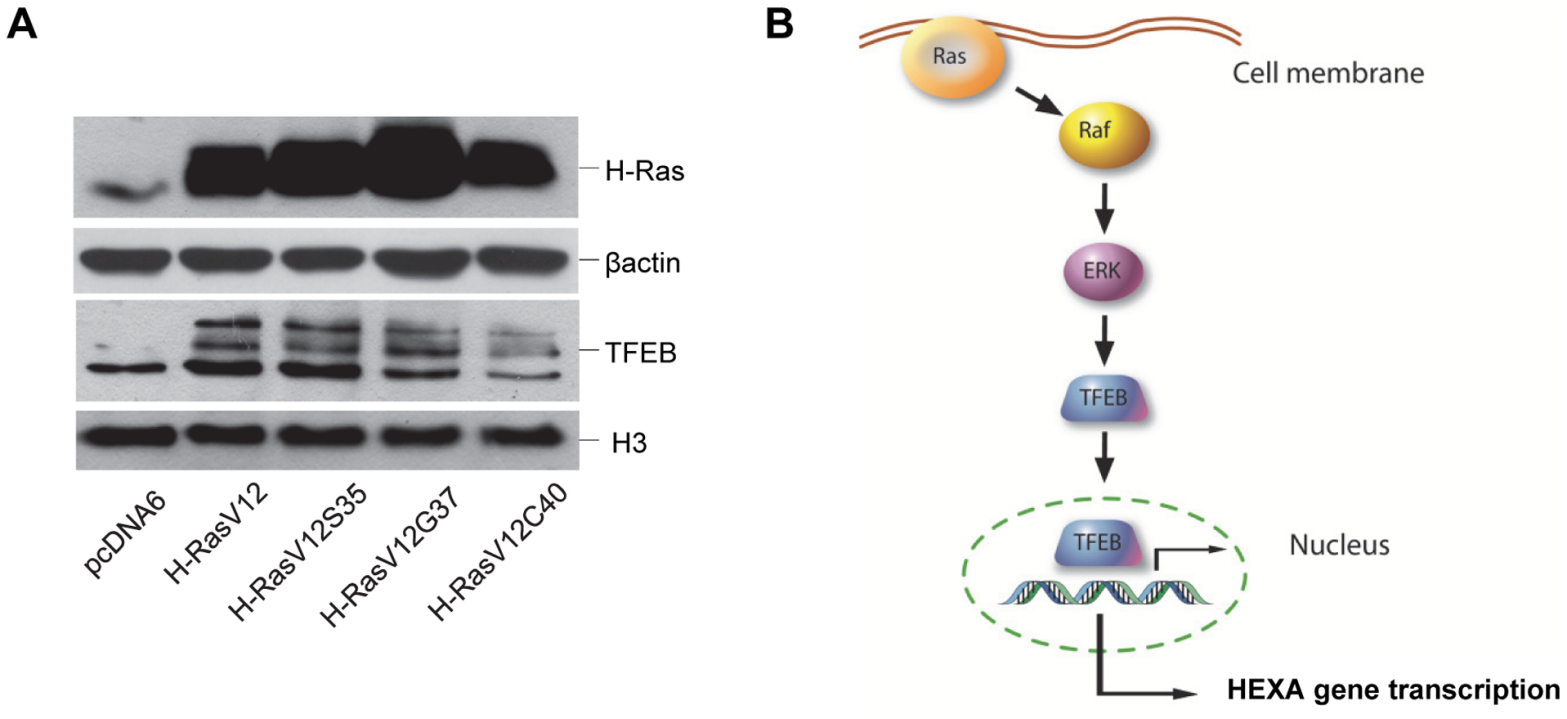
Immunoblotting analysis of TFEB expression in HuDe fibroblasts transfected with H-Ras mutants. A, Cytoplasm extracts were tested with anti-H-Ras antibody. As internal control an anti-β actin antibody was used. Nuclear extracts were tested with an anti-TFEB antibody. As internal control, an anti-H3 histone antibody was used. B, Model of lysosomal HEXA gene regulation by TFEB through a Raf/ERK dependent pathway.

On the basis of the above results, an increased nuclear localization of TFEB in H-RasV12 expressing cells could be hypothesized, so we analyzed by immunoblotting the expression of endogenous TFEB in HuDe cells transfected with H-Ras mutants. As shown in Fig. 8A, H-RasV12 expressing fibroblasts showed higher levels of nuclear TFEB with respect to mock transfected cells. Moreover, the increased nuclear translocation of TFEB was mediated by Raf/ERK pathway, as shown by H-RasV12S35 mutant, thus correlating with the increased enzymatic activity of β-hexosaminidase isoenzymes and the increased level of HEXA and HEXB transcripts.

## Discussion

Lysosomal enzymes have been often implicated in senescence and several studies demonstrated that lysosomal alterations are common in senescent cells [36]. Here we provide the evidence that lysosomal enzyme alterations are not only a prominent feature of senescence induced by oncogenic H-Ras, but they are also associated with an increased activation of TFEB transcription factor, a novel mTORC1 effector implicated in lysosome biogenesis and autophagy [37]. Specifically, β-hexosaminidase is an acidic glycohydrolase that cleaves off terminal β-linked GlcNAc or GalNAc residues from glycoconjugates. Its altered expression has been often associated with cancer [23], [24] and we previously showed that β-hexosaminidase isoenzymes pattern in leukaemic cells is significantly different from that in their normal counterparts, being characterized by the presence of Hex S, a homodimer of the α-subunit encoded by HEXA gene [18].

In our cell model the expression of constitutively active H-Ras did not result in an increase in intracellular β-hexosaminidase isoenzymes level, but rather an increase in Hex A and Total Hex enzymatic activity in cell culture medium. Two other lysosomal glycohydrolases, α-mannosidase and β-galactosidase, showed a significant increase of enzymatic activity in cell extracts, but it was not possible to assess their level in cell medium, because of the very low specific activity (data not shown). These results suggest that during OIS, lysosomal glycohydrolases are up-regulated by a mechanism that is general and not enzyme-specific, even if enzyme-specific differences could be detected (i.e. β-hexosaminidase enzymatic activity was not increased in cell extracts as the enzymatic activity of α-mannosidase and β-galactosidase), and this could probably reflect differences in the intracellular trafficking and stability of each protein.

The increased enzymatic activity of β-hexosaminidase isoenzymes suggested a possible up-regulation at the transcriptional level. This was confirmed by qRT-PCR analysis, which showed a significant increase of both HEXA and HEXB gene transcripts upon H-RasV12 expression. In addition, the analysis of the H-RasV12S35 mutant demonstrated that the transcriptional up-regulation is mediated by the Raf/ERK pathway. This result correlates with the enzymatic activity enhancement in cell culture medium observed for the same mutant. The demonstration that HEXB transcript was increased to a higher level with respect to HEXA transcript, while Total Hex enzymatic activity was increased to a lower level with respect to Hex A enzymatic activity, could be explained by differences in the stability of transcripts.

HEXA and HEXB promoters have been preliminarily characterized [34] but there were no evidence of protein binding to promoter active segments suggesting a possible molecular mechanisms at the basis of their transcriptional up-regulation. Therefore we investigated gene regulatory mechanisms underlying HEXA and HEXB genes expression. Deletion constructs showed that the HEXA promoter is mainly regulated by a short sequence located at −78/−100 position with respect to the first ATG, while that HEXB gene promoter is primarily regulated by a sequence located at −83/−134 position with respect to the first ATG. These results were in agreement with previous observations [34] and restricted promoter active sequences to shorter segments. In addition, they were consistent with similar findings about the promoters of other lysosomal enzymes, such as human N-acetylgalactosamine-6-sulfatase gene [32] and α-mannosidase gene [38], which are also characterized by short promoter active sequences (less than 100 bp segments of the 5′ flanking region). The occurrence of an E-box in the promoter active sequence of the HEXA gene suggested that this could be the main regulatory element controlling its expression. Mutational analysis confirmed a major role of this E-box in the regulation of HEXA promoter. Co-expression of H-Ras mutants with an HEXA promoter construct mutated in the E-box region clearly confirmed that the up-regulation of HEXA promoter activity driven by H-RasV12 is mediated by protein binding to this region. In the case of HEXB, at least two regulatory regions were identified, the first between −83 and −105, and the second between −105 and −134. This second region includes an E-box, but taken together, these results indicate a more complex regulation of this promoter.

The E-box found in human HEXA promoter −100/−78 segment is a known target site for basic bHLH transcription factors and overlaps a CLEAR motif (Fig. 6A). The CLEAR motif is a palindromic 10 bp motif highly enriched in lysosomal gene promoters which regulates the transcription of lysosomal genes through the binding of TFEB, a member of the Myc-related, bHLH leucine-zipper family of transcription factors [39]. Interestingly, we have observed that TFEB activation promotes the recruitment of lysosomal glycohydrolases β-hexosaminidase and β-galactosidase to the plasma membrane [21]. The HEXA gene promoter sequence includes three CLEAR elements recognized by TFEB [35], but our data showed that only the element located at –90 bp with respect to the first ATG is relevant to drive gene expression in our cell model, although it is not possible to rule out a role for the two other CLEAR motifs in different pathological or physiological conditions. We clearly observed protein binding to the region −100/−78 of HEXA gene promoter and *in vitro* and *in vivo* analyses confirmed that the sequence was actually bound by TFEB. Besides, TFEB overexpression was clearly able to transactivate HEXA gene promoter active segment, and this ability was impaired when TFEB binding sequence was mutated. TFEB overexpression also induced an increase of Hex isoenzymes activity in human fibroblasts, both in cell extracts and culture medium, whereas TFEB knock down decreased Hex isoenzymes activity, in agreement with previous observations showing that TFEB is at the basis of the transcriptional regulation of lysosomal exocytosis [40]. Even if the CLEAR motif is usually bound by TFEB, we cannot exclude that other transcription factors normally binding to E-boxes could also potentially recognize this sequence in different pathological or physiological conditions. TFEB was found to specifically bind DNA in both homodimeric and heterodimeric form in association with TFE3, another member of the MiTF/TFE bHLH leucine zipper sub-family [41]. In addition, other transcription factors potentially binding to E-boxes and characterized by a bHLH leucine zipper motif include members of the Myc/Max/Mad family [42], which are involved in the pathogenesis of many cancers and whose stability is regulated by H-Ras [43]. Of consequence, their ability to transactivate lysosomal glycohydrolases gene expression could be relevant in pathological conditions such as cancer and should be further investigated. As HEXA gene expression is increased following H-RasV12 expression and is mainly regulated by TFEB, we eventually analyzed TFEB level in fibroblasts expressing constitutively active H-Ras mutants and clearly observed that TFEB nuclear localization is also increased. Moreover, this up-regulation is mediated by the Raf/ERK pathway. This result is in agreement with previous observations showing that TFEB activity is controlled by the p42 MAPK (ERK2) [44] pathway, which is in turn activated by H-RasV12 [27]. Our findings indicate that during senescence induced by oncogenic H-Ras, lysosomal enzymes expression may be up-regulated by TFEB through a Raf/ERK dependent pathway (Fig. 8B). Taking into account the pivotal role of TFEB in the regulation of lysosomal system, our results clearly suggest that TFEB activation is at the basis of a general mechanism to enhance lysosomal enzymes specific activity during OIS. TFEB was previously shown to up-regulate lysosomal function in different cell models, linking autophagy to lysosomal biogenesis. Besides, autophagy activation upon the induction of OIS was also reported [45], [46]. However, it remains unclear whether H-Ras activation can induce the up-regulation of lysosomal system not only in senescent cells but also in cell models characterized by their ability to escape OIS due to the ablation of tumor suppressor pathways, such as p19Arf-p53 and p16Ink4a-Rb. Further investigations in this direction could shed light on the complex and unclear relation between the autophagy-lysosomal pathway and oncogenic transformation.
